# SERIAL TRICHOTOMIZATION TO IDENTIFY UNSAFE DRIVERS: UPDATE FROM A PROSPECTIVE STUDY

**DOI:** 10.1093/geroni/igad104.1355

**Published:** 2023-12-21

**Authors:** Michel Bedard, Sacha Dubois, Hillary Maxwell, Stephanie Schurr, Bruce Weaver, Arne Stinchcombe

**Affiliations:** Lakehead University, Thunder Bay, Ontario, Canada; St. Joseph’s Care Group, Thunder Bay, Ontario, Canada; St. Joseph’s Care Group, Thunder Bay, Ontario, Canada; St. Joseph’s Care Group, Thunder Bay, Ontario, Canada; Lakehead University, Thunder Bay, Ontario, Canada; University of Ottawa, Ottawa, Ontario, Canada

## Abstract

Identifying older adults who may be unsafe to drive remains a difficult task. Gibbons et al. (2017, American Journal of Occupational Therapy) used serial trichotomization based on five cognitive tests to determine if drivers should: 1) continue driving, 2) undergo further evaluation, or 3) stop driving. The tests included the Trail-making-tests A and B (TMT-A/B), the clock-drawing test (CDT), the Motor-Free Visual Perception Test (MVPT), and the Montreal Cognitive Assessment (MoCA). Gibbons et al. relied on dual cut-off values to achieve 100% sensitivity and specificity (within their sample) to reduce false positives and false negatives that arise from using these tests in stand-alone fashion. We used the Gibbons et al. cut-off values prospectively on a cohort of 293 drivers (mean age = 66, SD = 14) referred for driving evaluations at a chronic care and rehabilitation hospital. Each driver completed the five tests. Trained occupational therapists (OTs) provided a recommendation to continue driving, undergo further evaluation, or stop driving. We examined congruence between the tests and the OTs recommendations. Weighted Kappas ranged from a low of .03 (95% CI = -.01 to .08) for the CDT, to a high of .53 (95% CI = .45 to .61) for the TMT-B. Using the same cut-offs, and serial trichotomization, the congruence with the final recommendations was moderate (k = .57, 95% CI = .49 to .66). These results remind us of the variability inherent in stand-alone cognitive tests and even within a serial trichotomization framework.

